# Sex differences in behavioural and neural responsiveness to mate calls in a parrot

**DOI:** 10.1038/srep18481

**Published:** 2016-01-04

**Authors:** Hiroko Eda-Fujiwara, Ryohei Satoh, Yuka Hata, Marika Yamasaki, Aiko Watanabe, Matthijs A. Zandbergen, Yasuharu Okamoto, Takenori Miyamoto, Johan J. Bolhuis

**Affiliations:** 1 Laboratory of Behavioral Neuroscience, Faculty of Science, Japan Women’s University, Tokyo, Japan; 2 Graduate School of Human Arts Sciences, University of Human Arts and Sciences, Saitama, Japan; 3 Department of Physiology, Kitasato University School of Medicine, Kanagawa, Japan; 4 Cognitive Neurobiology and Helmholtz Institute, Utrecht University, Utrecht, The Netherlands; 5 Department of Psychology, Japan Women’s University, Kanagawa, Japan

## Abstract

Vocalisation in songbirds and parrots has become a prominent model system for speech and language in humans. We investigated possible sex differences in behavioural and neural responsiveness to mate calls in the budgerigar, a vocally-learning parrot. Males and females were paired for 5 weeks and then separated, after which we measured vocal responsiveness to playback calls (a call of their mate versus a call of an unfamiliar conspecific). Both sexes learned to recognise mate calls during the pairing period. In males, but not females, mate calls evoked significantly fewer vocal responses than unfamiliar calls at one month after separation. Furthermore, in females, there was significantly greater molecular neuronal activation in response to mate calls compared to silence in the caudomedial mesopallium (CMM), a higher-order auditory region, in both brain hemispheres. In males, we found right-sided dominance of molecular neuronal activation in response to mate calls in the CMM. This is the first evidence suggesting sex differences in functional asymmetry of brain regions related to recognition of learned vocalisation in birds. Thus, sex differences related to recognition of learned vocalisations may be found at the behavioural and neural levels in avian vocal learners as it is in humans.

The capacity for vocal imitation learning is a prerequisite for the evolution of human spoken language (speech)^1^. This capacity is a rare trait in the animal kingdom, absent in non-human primates, but present in certain marine mammals and three avian taxa (songbirds, parrots, and hummingbirds)^1^,^2^. Thus, vocal learning in birds has become a prominent animal model for human speech acquisition^2^,^3^,^4^. In addition to changes in vocal production, auditory recognition learning enables complex communication in both humans and birds^3^. Furthermore, the brain regions of avian vocal learners involved in vocal production and auditory recognition are analogous to the brain regions that are important for producing and understanding speech in humans^2^,^3^,^4^. The premotor nucleus HVC (a letter-based name) of songbirds plays an important role in song production and sensorimotor learning (reviewed in^5^), and thus may be analogous to the network of brain regions centred around Broca’s area in the human frontal lobe^2^,^3^,^6^. Forebrain regions of songbirds, the caudomedial nidopallium (NCM) and the caudomedial mesopallium (CMM), are involved in auditory recognition and perception. As such, they are thought to be the avian equivalent of the human auditory association cortex in the temporal lobe, including Wernicke’s area^2^,^6^.

In many songbird species, there are sex differences in brain morphology and vocal behaviour^7^. Both behaviourally and neuroanatomically, the zebra finch (*Taeniopygia guttata*) is one of the most sexually dimorphic songbirds. Male zebra finches learn their vocalisations (both songs and calls), while females do not^8^. Consistent with sex differences in vocal behaviour, the HVC is larger in male than in female songbirds^9^. During development, both female and male zebra finches acquire a preference for the song of their father over unfamiliar conspecific song, which indicates that both sexes can learn to recognise tutor song^10^. In adult zebra finches, females do not differ from males in learning to discriminate between two songs of unfamiliar males^11^. In zebra finches, memory-related neuronal responsiveness to song was found in the CMM in females, and in the NCM in males^12^,^13^,^14^,^15^,^16^,^17^.

Parrots (of both sexes), can imitate vocalisations throughout life^18^,^19^,^20^,^21^,^22^,^23^,^24^. Parrots resemble humans in that they use their tongue to articulate^25^ and they can synchronize their movement to a musical beat^26^,^27^,^28^. Despite these intriguing behavioural parallels, there have been few studies investigating whether there are sex differences in vocal production and recognition learning in parrots. The budgerigar (*Melopsittacus undulatus*) is a parrot species of which both sexes produce individually distinctive contact calls (Fig. 1). At any given point in time, a bird may have a repertoire of one to several different types of contact calls^18^,^21^. Although the capacity for vocal imitation is present in male and female adult budgerigars, there are sex differences in call production learning. Male budgerigars learn new calls more quickly than females and during pair bond formation males learn to produce the calls of females and thus create a new call type, but not vice versa^20^,^21^. The budgerigar, like songbirds, has specialised, discrete brain regions for vocal production, auditory perception and learning^29^,^30^,^31^,^32^,^33^. The central nucleus of the lateral nidopallium (NLC) of the budgerigar corresponds to the songbird HVC, a song system nucleus that plays an important role in song production and sensorimotor learning^29^,^31^,^33^. Consistent with sex differences in vocal production learning, it was found that the NLC is significantly larger in males than in females^34^. A recent study reported that, unlike other vocal learning bird lineages, the parrot song system has core and shell regions^35^. Although the area ratios of the core and shell regions of the NLC differ among nine parrot species including the budgerigar^35^, it remains to be explored whether such a relative size difference exists between the sexes in the budgerigar.

There has been little investigation of sex differences in recognition learning of the budgerigar. After experience during the pairing period, female budgerigars come to respond to the call of their mate more than to a call of an unfamiliar male^36^. Not much is known about males in this respect, but a preliminary study reported that two of three male budgerigars tested responded to the call of their mate more than the calls of other familiar budgerigars^37^. To investigate possible sex differences in recognition learning, we carried out two experiments, using the same paradigm as that in our previous study of female budgerigars^36^. We examined vocal responses of males in experiment 1, as those of females were examined previously^36^. Budgerigar males change vocal behaviour depending on stages of courtship and reproduction^38^. We then examined vocal responses of both sexes in experiment 2, keeping the rate of development of the pair bond the same between males and females and allowing for a more stringent test of sex differences.

In addition to evidence for differential neuronal activation for conspecific vocalisations (i.e., song and call) between sexes in the NCM and the CMM of songbirds^12^,^13^,^39^,^40^,^41^,^42^, there is growing evidence for lateralisation of neural activation in relation to song recognition in the NCM and the CMM of songbirds^43^. This is reminiscent of speech- and language-related neuronal activation in humans, which is mostly lateralised to the left hemisphere^15^,^16^,^43^. However, sex differences have not been reported about functional asymmetry in higher-order auditory regions of songbirds^43^. In the budgerigar, there have been few studies investigating neuronal activation to conspecific vocalisations in the NCM and the CMM^29^,^32^, and has been no study examining brain lateralisation in response to conspecific vocalisations in parrots. Here, we investigated possible sex differences and/or lateralisation of neuronal activation, measured as the expression of Zenk (the protein product of the immediate early gene *ZENK*, an acronym of *zif-268*, *egr-1*, *ngf-Ia*, and *krox-24*), in response to mate calls in the NCM and the CMM of male and female budgerigars.

## Results

### Sex differences in behavioural responsiveness to mate calls

Figures 2a and 3a depict the experimental design. The call-response ratio significantly greater than 0.5 indicates that mate calls evoke more calls than unfamiliar calls. In contrast, the ratio significantly smaller than 0.5 indicates that mate calls evoke fewer calls than unfamiliar calls.

#### Experiment 1

The call-response ratio changed among months (i.e., 0, 1, and 5 months after pair separation, repeated-measures ANOVA: F_2,18_ = 15.622, P < 0.001; Fig. 2b). *Post-hoc* Bonferroni tests revealed significant differences in call-response ratios between m1 and both m0 (P < 0.01) and m5 (P < 0.01). Males responded to mate calls significantly less than to unfamiliar calls, i.e., their call-response ratio was significantly different from chance (0.5) at m1 (One-sample t test: t_9_ = −7.489, P < 0.001). However, the call-response ratio was not significantly different from 0.5 at m0 (t_9_ = −1.121, P > 0.2) or m5 (t_9_ = −0.059, P > 0.8).

We compared means of the total number of calls (i.e. vocal activity level) among months with a repeated-measures ANOVA. There was no significant change with time in the total number of calls during playback (repeated-measures ANOVA: F_2,18_ = 3.090, P = 0.070).

*Experiment 2*. Before the start of pairing period, both sexes preferred neither of the calls presented, that is, call-response ratio was not significantly different from chance (0.5) in males (t_10_ = −0.063, P > 0.8) and in females (t_5_ < 0.001, P > 0.8; Fig. 3b). Immediately after separation, however, both sexes responded to the mate call significantly more than to the unfamiliar call (One-sample t test; Female: t_6_ = 2.758, P < 0.05; Male: t_10_ = 2.418, P < 0.05). Thus, both sexes learned to recognise mate call during the pairing period. A repeated-measures ANOVA with Sex as independent factor and Time (i.e. before the pairing period and 0 month after pair separation) as repeated factor revealed a significant interaction between Sex and Time (F_1,14_ = 5.929, P = 0.029) and a significant effect of Time (F_1,14_ = 12.396, P = 0.003) on the call-response ratio for mate calls. These results show sex differences in vocal response for mate calls. A subsequent repeated-measures ANOVA with Sex as independent factor and Time (i.e. 0 and 1 months after pair separation) as repeated factor revealed a significant effect of Sex (F_1,13_ = 13.945, P = 0.003) and a significant effect of Time (F_1,13_ = 16.373, P = 0.001) on the call-response ratio for mate calls. There was no significant interaction between Sex and Time. Similarly to the first experiment, males showed a significantly less response for the mate call at one month after separation (One-sample t test: t_10_ = −2.235, P < 0.05). Males and females showed no differences in vocal activity levels (means of the total number of calls+ s.e.m.; Female at m0: 222 ± 91; Male at m0: 248 ± 71; Female at m1: 279 ± 166; Male at m1: 165 ± 50), that is, a repeated-measures ANOVA with Sex as independent factor and Time (i.e. 0 and 1 months after pair separation) as repeated factor revealed no significant effect of Sex, Time or interaction between Sex and Time on the total number of calls during playback.

#### Pair bonding

In both experiments, the mate-specific behaviour increased during the period of pairing (repeated-measures ANOVAs for 2, 15, and 35 days after the pairs were placed into breeding cages: experiment 1: F_2,18_ = 4.018, P = 0.036; experiment 2: F_2,22_ = 5.863, P = 0.009). *Post-hoc* Bonferroni tests revealed significantly more mate-specific behaviour on day 35 than on day 2 (P < 0.01), but no difference between day 2 and 15 in experiment 1, and more mate-specific behaviour on day 15 than on day 2 (P < 0.01), but no difference between day 2 and 35 in experiment 2. Furthermore, eight of 12 females laid eggs by day 35 after the start of pairing in experiment 2, but only one of 10 females in experiment 1. Thus, the development of pair bonds, assessed by the mate-specific behaviour and the number of eggs, was slower in experiment 1 than experiment 2.

### Sex differences in neural responsiveness to mate calls

Figure 4 shows representative photomicrographs of Zenk-immunoreactive (-IR) cell nuclei in the three sampled brain regions in both sexes. The mean number of Zenk-IR cells per square millimetre was calculated in the CMM, the NCM, and the hippocampus (Fig. 5). Overall, a two-way ANOVA (Sex and Stimulus as factors) showed a significant effect of Sex (F_1,19_ = 6.586, P = 0.019). We then analysed the results for the two sexes separately. Repeated-measures ANOVAs revealed a significant effect of Brain Region on the number of Zenk-IR cells in females (F_2,33_ = 26.251, P < 0. 001) and in males (F_2,30_ = 15.389, P < 0.001). Because there was a significant effect of Brain Region, the results were analysed for each of the three brain regions in each sex.

In females, a repeated-measures ANOVA revealed a significant effect of Stimulus on the number of Zenk-IR cells for the CMM (F_1,10_ = 6.916, P = 0.025), but not for the NCM or the hippocampus (Fig. 5a). There was no significant effect of Hemisphere or interaction between Stimulus and Hemisphere for each of the three brain regions.

There was no significant effect of Stimulus or Hemisphere in the CMM, the NCM, or the hippocampus of males (Fig. 5b). However, there was a significant interaction between Stimulus and Hemisphere for the CMM (F_1,9_ = 8.259, P = 0.018), but not for the NCM or the hippocampus. These results suggest lateralised neuronal activation in response to the mate call in the CMM of males. Data were normalized to the mean number of Zenk-IR cells in the appropriate silent control group (the normalized number of Zenk-IR cells, mean ± s.e.m.: 1 ± 0.120 in the left Silence group; 0.899 ± 0.074 in the left Mate group; 1 ± 0.186 in the right Silence group; 1.295 ± 0.112 in the right Mate group). A repeated-measures ANOVA revealed no significant effect of Stimulus, but a significant effect of Hemisphere (F_1,9_ = 8.242, P = 0. 018) and a significant interaction between Stimulus and Hemisphere (F_1,9_ = 6.868, P = 0.028) on the normalized Zenk-IR cells for the CMM of males. There was no significant interaction between Stimulus and Hemisphere, or no significant effect of Hemisphere on the normalized Zenk-IR cells for the CMM of females.

## Discussion

Female budgerigars showed significantly stronger behavioural response for mate calls over calls of unfamiliar males, which is consistent with a previous study^36^. In contrast, male budgerigars showed a significantly weaker behavioural response to the calls of their mates. This indicates that the males acquired an auditory memory of their mate call during the 5 weeks of pairing period and could distinguish mate calls from unfamiliar calls. These findings demonstrate that both sexes can learn to recognise mate calls during the pairing period, despite sex differences in behavioural responsiveness to mate calls in the budgerigar.

The male budgerigars showed weaker behavioural response for mate calls after one month of separation in experiment 1 and 2. Meanwhile, on separation from the mates we observed differences in male response patterns between experiment 1 and 2. This difference between the two experiments may be explained by the difference in the rate of pair bond development, which was assessed by mate-specific behaviour and the number of eggs in the present study. A previous study in the budgerigar showed the change in one aspect of male vocal behaviour with pairs proceeding through stages of courtship and reproduction^38^. After eggs are laid and male budgerigars begin to feed their mates, call similarity of members of a pair decrease, indicating that males change the call repertoire depending on stages of reproduction^38^. In the present study, the development of pair bonds was faster in experiment 2 than in experiment 1. After egg laying the males in experiment 2 might have increased call response to mate calls.

We found stronger behavioural responsiveness for mate calls over unfamiliar calls in females than in males in experiment 2. This sex difference in the magnitude of the behavioural response to mate calls cannot be explained by the difference in the rate of pair bond development between the sexes, because we directly compared female and male budgerigars in experiment 2. Is the sex difference in behavioural responding explained by the sex difference in recognition learning? Conceivably, variation in call-response ratio found here could be the result of motivational factors as well as recognition learning. Male zebra finches were found to show preferential responsiveness to mate calls over familiar female calls in the presence of an established male-female pair, but not in the presence of unmated conspecifics^44^. Social context may alter motivational factors and then vocal behaviours in male budgerigars. Since we cannot exclude a motivational explanation, sex differences in the strength of recognition learning remains to be concluded.

Marler^45^ discussed the value of including bird calls in the sensory physiology of signal perception. However, so far, neuronal activation in response to call stimuli has not been investigated in the NCM or the CMM of the budgerigar, although a part of Field L as identified by Brauth *et al.*^30^ was shown to respond to call stimuli, and corresponds to the NCM (but not the CMM) as the term is used in the other studies^29^,^32^,^46^ and in the present paper. Neuronal activation in the CMM in response to mate calls differed between male and female budgerigars, with females showing greater activation. The present results are the first suggesting sex differences in neuronal activation by conspecific vocalisation of parrots (Psittaciformes). Generally in birds and mammals, sex differences in brain functions can be explained not only by sex steroids secreted from the gonads, but also by other mechanisms independent from gonadal hormones (i.e., brain-derived steroids and sex chromosomes in the brain)^47^. In the songbird NCM, estrogens rapidly produced in the brain are important for auditory processing of conspecific song^48^ and thus can be one of mechanisms underlying sex differences in auditory processing. In the budgerigar, estrogen receptors are not expressed in the NCM^49^. As for the CMM, the expression of estrogen receptors has never been reported, or extensively studied in the budgerigar. To test whether brain-derived estrogens contribute to sex differences reported in the present study, it would be interesting to investigate the expression of estrogen receptors and the estrogen-synthetic enzyme aromatase in the CMM of the budgerigar.

The results of a number of songbird studies have suggested that neuronal activation of forebrain auditory regions in response to conspecific vocalisations might reflect stimulus salience^41^,^42^,^50^. A series of studies on other vertebrate species also support the view that molecular neuronal activation in response to conspecific vocalisations reflects behavioural salience of stimuli. For example, in a well-studied species, the túngara frog (*Physalaemus pustulosus*), there is greater neuronal activation, measured as expression of the immediate early gene *egr-1* (also known as *ZENK*), in response to behaviourally salient conspecific calls compared to non-salient heterospecific calls in the auditory brainstem and its forebrain targets^51^. In the present study, when overall activation from both hemispheres was examined, there was greater neuronal activation in response to mate calls than to silence in female budgerigars, but not in males. In the behavioural test at one month after separation, which was conducted before the neural analysis, mate calls evoked significantly fewer vocal responses than unfamiliar calls in males, but not in females. Thus, the salience of mate calls might be different between the sexes, resulting in sex differences in neuronal activation to mate calls as reported in this study.

Alternatively, it may be that the increased neuronal activation in the CMM is explained by stimulus novelty. *ZENK* expression in the NCM of songbirds is thought to be strongly dependent on the novelty of a stimulus and habituates rapidly when stimulus novelty decreases^52^,^53^. Field L complex of the budgerigar exhibits Zenk protein induction in response to stimulation with an unfamiliar contact call^30^. Such Zenk protein induction is attenuated when the budgerigars are familiarized with a previously unfamiliar contact call through repeated playbacks of the call for 24 hours^30^. In the present study, the stimulus mate calls which were not novel stimuli lead to increased neuronal activation in the CMM of female budgerigars. The increased neuronal activation in the present study could be explained by familiarity. The present experiment was designed to explore possible sex differences in higher auditory regions of the budgerigar. We used birds in silence as control, but not birds exposed to unfamiliar calls, as neuronal activation to contact calls in the budgerigar CMM has not been investigated, and our sample size was limited. To address the issue of familiarity explicitly, responsiveness to mate calls should be compared with responsiveness to unfamiliar calls.

There was a significant interaction between Stimulus and Hemisphere on Zenk-IR cells in the CMM of male budgerigars. This interaction suggests lateralised neuronal activation resulting from exposure to mate calls in the CMM of males. There is considerable evidence for lateralisation of neural activity in response to conspecific vocalisation in songbirds^43^,^54^. In contrast, so far there is little evidence for brain lateralisation in response to conspecific vocalisation in parrots (Psittaciformes)^54^. There is indirect evidence of perception-related lateralisation (eye lateralisation) in Australian parrots^55^. The present results are the first suggesting perception-related lateralisation in the brain of parrots. As for hemispheric asymmetries for recognition of learned vocal signals in birds, a pioneer study in zebra finches suggested that the two hemispheres of songbird forebrains recognise and process conspecific vocal signals differently^56^. Subsequent songbird studies have shown functional hemispheric asymmetries in higher auditory brain regions, including the NCM and the CMM^15^,^48^,^52^,^57^,^58^,^59^.

Vocalisation in songbirds and parrots has become a prominent model system for speech and language in humans^60^. In a parrot, we found that there are sex differences in responsiveness to mate calls at behavioural as well as neural level. In male budgerigars, but not in females, we found right-sided dominance of neuronal activation to mate calls in the CMM. This is the first evidence suggesting sex differences in functional asymmetry of brain regions related to recognition of learned vocalisation in birds. The CMM is, in a functional sense, considered to be analogous to human Wernicke’s area^2^,^6^. In humans, sex differences have been observed in performance on language-related tasks and in the neural bases of language. Functional hemispheric asymmetries have been found in human cortical regions involved in perception of speech (reviewed in^61^). It has been hypothesised that human language functions are more strongly lateralised to the left hemisphere in males than in females (reviewed in^62^), although the debate about sex differences in lateralisation of language is still ongoing^62^,^63^. Sex differences in neuronal activation to conspecific vocalisations have been reported in the higher auditory regions of songbirds^12^,^13^,^39^,^40^,^41^,^42^ and a parrot in this study. Recently Yoder *et al.*^64^ reported sex differences in the processing of learned vocalisations in the NCM of the zebra finch, and that the hormone estrogens affect the processing. However, estrogen receptors are not expressed in the NCM of the budgerigar^49^, suggesting a mechanism underlying sex differences in the auditory processing different from that in the zebra finch. Thus parrots as well as songbirds may offer an opportunity to study the biological basis of sex differences in speech and language.

## Methods

### Subjects and housing

Budgerigars (22 males and 22 females) were obtained from a local supplier. All birds were kept in a controlled environment suitable for breeding (23 ± 3 °C and a light cycle of 14:10 h light:dark) throughout the study, as described previously^36^. Food, which consisted of a commercial seed mixture, and water were provided *ad libitum*. Cuttlebone was provided during the period when birds were housed in pairs.

We used 10 males and 10 females in experiment 1, and 12 males and 12 females in experiment 2. Experiments 1 and 2 were conducted with different birds. Birds were initially housed singly and their vocalisations were recorded. After recording of all birds was completed, pairing was initiated. After the pairing period of 5 weeks, each bird was isolated from the mate and kept in an individual cage without any subsequent auditory/visual stimulation from the mate. Because males and females were housed in separate rooms after the pairing period, each bird received auditory/visual stimulation from other birds from the same sex, but not from any other birds of the opposite sex. We quantified the amount of the mate-specific behaviour (the sum of occurrences of allopreening and courtship feeding) on days 2, 15, and 35 after the pairs were placed into breeding cages to assess the development of pair bonds, as described previously^36^. The number of eggs in the nestboxes was checked on day 35 (see the supplementary information for details on housing and behavioural observations).

### Recording and analysis of contact calls

**Figure 1:**
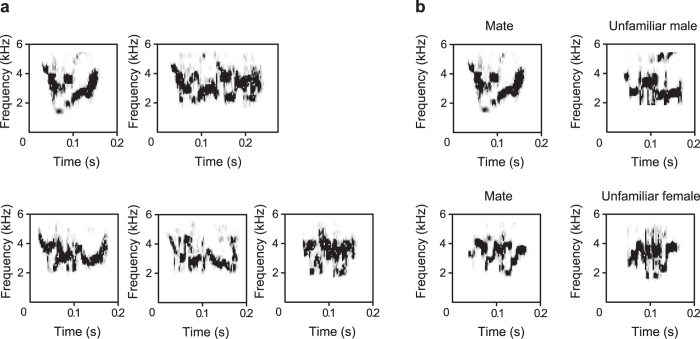
Spectrograms of contact calls in the behavioural test. (**a**) The call repertoire consisting of 5 types in a male budgerigar. Of all call types in the repertoire, one call type occurs most frequently and is therefore termed ‘the dominant contact call’ (upper left for this male). The dominant call recorded before pairing was used as stimulus mate’s call in the behavioural test. (**b**) Calls were presented to a female (upper) and a male (lower). We measured the number of calls emitted by each bird in response to a call of the mate (left) and a call from an unfamiliar bird of the opposite sex (right).

Contact calls are strongly frequency-modulated, mostly within the range of approximately 2–4 kHz (Fig. 1). The budgerigar contact call does not show marked sexual dimorphism in acoustic structure. We recorded and analysed the call repertoires of all birds prior to pair formation. The methods used to record and analyse contact calls were similar to those described in previous studies^21^,^36^. We recorded at least 80 contact calls per bird. After classifying contact calls into call types, we paired birds disassortatively with respect to calls, that is, pair members did not have similar calls at this time, as determined by visual inspection of spectrograms. All the call types of each male were compared with those of his mate, by visual inspection of spectrograms. The observer, who classified calls, rated the degree of similarity as 1 (no similarity), 2 (fair similarity) or 3 (good similarity). All comparisons were rated as 1 (see the supplementary information for details on construction of stimuli and recording of contact calls).

### Behavioural test

In a 2 h trial, tested birds were presented with two call stimuli: that is, a series of the mate’s call and a series of the call from an unfamiliar bird of the opposite sex were broadcast alternately (series duration = 10 s, with one call every 2 s; 2 s interval between series), as described previously^36^ (Fig. 1). By visual inspection of spectrograms, we compared the stimulus calls with all the calls in the repertoire of the birds being presented with the stimuli. We assigned to each bird two stimulus calls: a mate’s call and an unfamiliar call, under the condition that both the mate’s and unfamiliar calls were scored ‘1’ (no similarity) on our ordinal scale of similarity, compared to all of the call types in each subject bird. Female budgerigars show significantly stronger behavioural responsiveness to mate calls than to calls of unfamiliar males at one month after separation from their mates, but cease to show this responsiveness at 6 months after separation^36^. To examine whether this responsiveness is maintained and eventually extinguished also in males, we repeatedly conducted behavioural tests for each bird as mentioned below.

**Figure 2:**
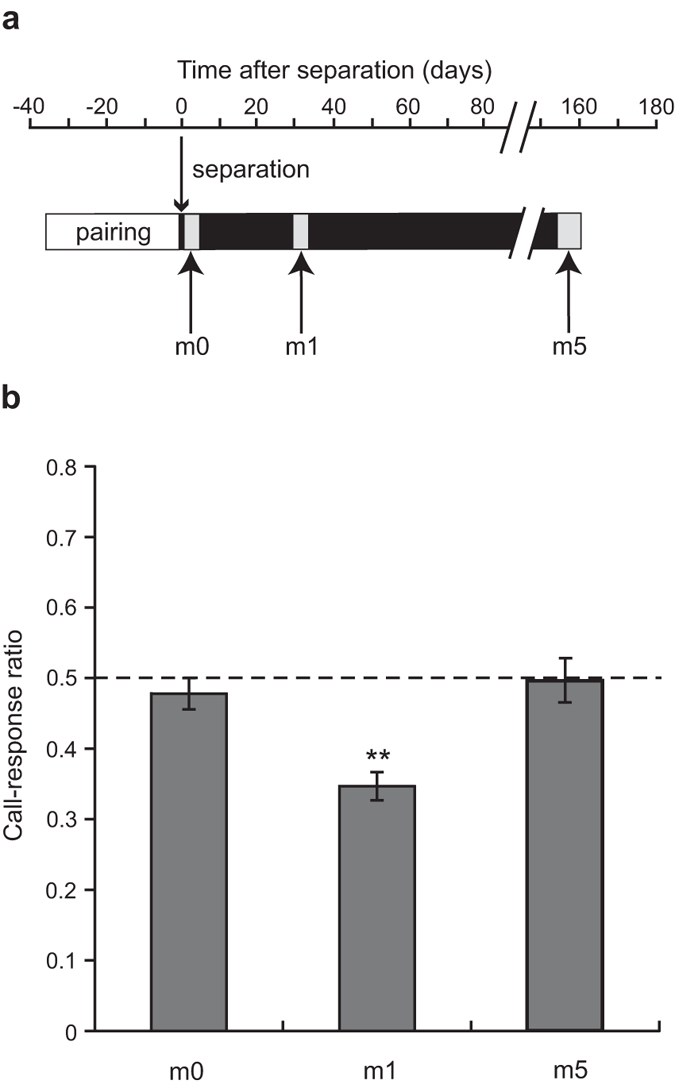
Call response in experiment 1. (**a**) Before the behavioural tests, males and females were paired for 5 weeks and then separated, without auditory/visual stimulation from the mate. Birds were unfamiliar with their mate prior to the pairing but they could memorize their mate’s call during the pairing period of 5 weeks. Each male was given three behavioural tests at 0 month (m0), 1 month (m1) and 5 months (m5) after separation. (**b**) Mean (+ s.e.m.) call-response ratios in experiment 1. The call-response ratio was calculated for each individual as the number of calls in response to the mate’s call divided by the grand total (i.e., the number of calls to the mate’s call and that to the unfamiliar call). Asterisks denote a significant difference from chance level (dashed line). The call-response ratio at m1 was significantly lower than 0.5 (**P < 0.01), indicating that males responded to mate calls less than to unfamiliar calls.

For each of 10 males of experiment 1, we conducted three behavioural tests at days 4 ± 4, 32.5 ± 1.5 and 161 ± 2 after separation, which were designated ‘0 month (m0)’, ‘1 month (m1)’ and ‘5 months (m5)’ (Fig. 2). Each stimulus pair was used repeatedly in the three tests for one male subject. We counted the calls produced by each male during the broadcast of the stimuli, as described previously^36^. In brief, each male was subjected to two 2 h trials in two successive days (4 h total per subject). Throughout 4 h recording, we counted the number of calls during each series of the mate’s call and counted that during each series of the call from an unfamiliar bird. The sample size was 10 males in three tests (i.e. m0, m1 and m5).

**Figure 3:**
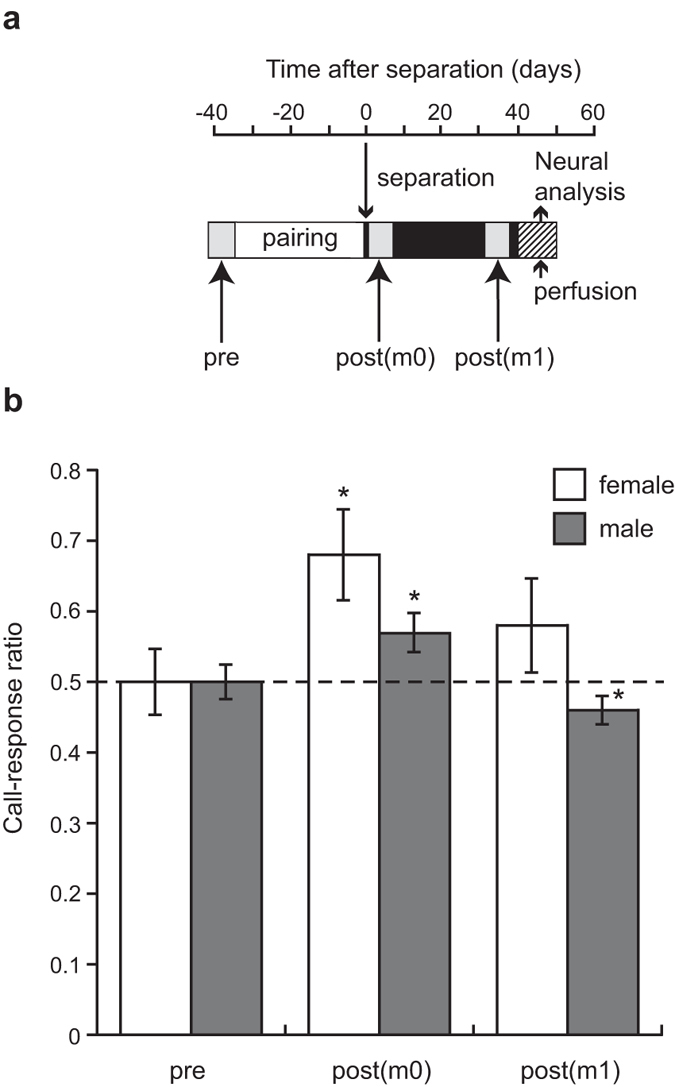
Call response in experiment 2. (**a**) Each bird was given three behavioural tests, before pairing and at 0 month and 1 month after separation, which are designated ‘pre’, ‘post(m0)’ and ‘post(m1)’, respectively. (**b**) Mean (+ s.e.m.) call-response ratios in experiment 2. Asterisks denote significant differences from chance level (dashed line) at m0 and m1 (*P < 0.05). Females showed a significantly greater response ratio to mate calls than males on separation and at one month after separation.

For each of 12 males and 12 females of experiment 2, we conducted three behavioural tests at days 38.5 ± 3.5 before separation and at days 3.5 ± 3.5 and 35 ± 5 after separation, which are designated ‘pre’, ‘post(m0)’ and ‘post(m1)’, respectively (Fig. 3). The procedure was identical to that in experiment 1. Female behaviours related to pairing are affected by call similarity in the budgerigar^38^. As mentioned above, we compared the stimulus calls with all the calls in the repertoire of the birds being presented with the stimuli. Then in experiment 1 we assumed that the call-response ratio was not different from 0.5 (chance level) before pairing in males. In order to confirm this assumption, for both sexes in experiment 2, we conducted behavioural test before pairing for possible effects of call similarity. We could not record the vocal activity of four females because of technical problems. There were no vocal responses from two females in the pre test, from one female in the post(m0) test, or three females in the post(m1) test. Therefore, in females the sample size was six in the pre test, seven in the post(m0) test and five in the post(m1) test. There were no vocal responses from one male in the pre test, in the post(m0) test, or in the post(m1) test. In males, the sample size was 11 in three tests, that is, pre, post(m1) and post(m1) tests (see the supplementary information for details on behavioural analyses).

### Reexposure for neural analysis

After the preference test at post(m1) in experiment 2, each bird was placed in a cage in a sound-attenuating chamber equipped with a speaker (AS-5; Kenwood Corp., Tokyo, Japan) for at least 12 h prior to the start of stimulus presentation. Then, each bird was exposed to a recording of the mate’s call (group Mate; n = 7 females, 7 males) or kept in silence (group Silence; n = 5 males, 5 females). On the day of (re-)exposure, lights were switched on at 8:30 AM as usual. Subsequently, lights were switched off 15 min before the onset of playback, which started at 10:45 AM. During playback, birds were kept in darkness to keep movement that could induce *ZENK* expression to a minimum and to prevent the birds from vocalising, as described previously^46^. During playback, each bird was exposed to the repetition of a single contact call (0.5 calls per sec) at regular intervals for 30 min. As stimuli for exposure, we used the same stimulus calls of seven males and seven females that were used in the behavioural tests of experiment 2. Mate calls were broadcast at the peak value of 80 dB SPL, measured 20 cm away from the speaker. The birds remained in darkness for 1 h after the end of call playback, when they were sacrificed. We monitored vocal behaviour of 24 birds during 30 min of playback. We adopted the criterion similar to that by Gobes *et al.*^39^ to exclude cases in which songs were produced or more than 5% of calls broadcasted from the speaker were produced by the subject. One male produced calls and songs and was excluded for the further analysis.

### Immunocytochemistry

One hour after the end of exposure to the stimulus, the birds were given an overdose of sodium pentobarbital (Nembutal) and subsequently perfused intra-cardially with saline and a fixative (4% paraformaldehyde in PBS). Frontal frozen brain sections were processed for Zenk-IR cell nuclei by immunocytochemistry, as described previously^32^,^46^. We processed all the tissues in four immunocytochemistry batches, counterbalancing the four treatment groups within each.

### Image analysis

**Figure 4:**
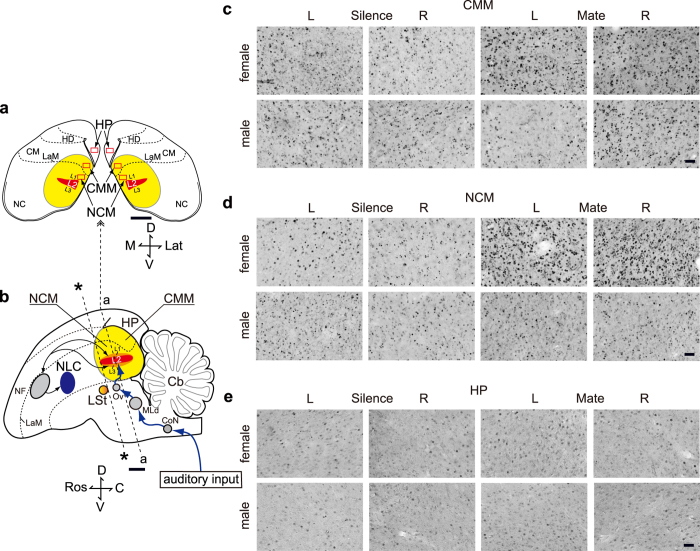
Neuronal activation in the budgerigar brain. (**a**) Coronal sections cut at level “a” in (**b**). Rectangles in the both side of sections represent the counting frames. (**b**) A schematic drawing of a parasagittal plane. The most caudal part of the LSt was defined as coordinates zero (*). Yellow regions in (**a**,**b**) and blue arrows in (**b**) indicate the caudomedial pallium and a primary auditory pathway, respectively. Lat, Lateral; Ros, Rostral; D, Dorsal; V, Ventral; C, Caudal; M, Medial. (**c**–**e**) Photomicrographs of coronal sections of the budgerigar brain showing Zenk immunoreactivity. Scale bar represents 1 mm in (**a**,**b**) and 50 μm in (**c**–**e**). Abbreviations; Cb, Cerebellum; CM, Caudal mesopallium; CMM, Caudomedial mesopallium; CoN, Cochlear nucleus; HD, Densocellular part of the hyperpallium; HP, Hippocampus; L1, L2, L3, Subdivisions of Field L complex; LaM, Mesopallial lamina; LSt, Lateral striatum; MLd, Dorsal part of the lateral mesencephalic nucleus; NC, Caudal nidopallium; NCM, Caudomedial nidopallium; NF, Frontal nidopallium; NLC, Central nucleus of the lateral nidopallium; Ov, Nucleus ovoidalis of the thalamus.

**Figure 5:**
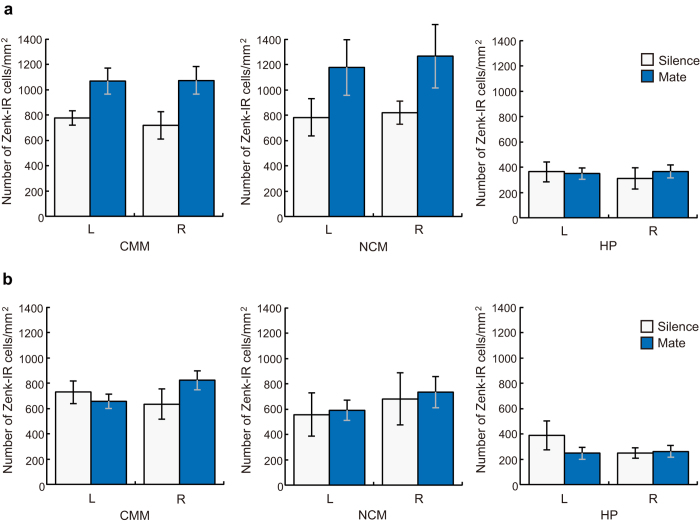
Neuronal activation in response to mate calls. Mean (+ s.e.m.) number of Zenk immunoreactive cells per square millimetre in the CMM, the NCM and the hippocampus (HP) in females (**a**) and in males (**b**). In males, there was a significant interaction between Stimulus and Hemisphere for the CMM, suggesting lateralised neuronal activation in response to the mate’s call in the CMM (see Results for statistics).

In our previous paper we sampled two regions within the NCM at dorsal and ventral levels (dNCM and vNCM) and found the dNCM more related to auditory recognition^46^. Then, in the present study, we sampled dNCM for the NCM. As a control, we also sampled the hippocampus, as described previously^32^,^46^. We captured photomicrographs of the counting frames (290 μm × 450 μm) with a digital microscope (BZ-9000; KEYENCE, Japan) and counted the number of Zenk-IR cells, “blind” as to the experimental history of the subjects. We took a total of four photomicrographs from both hemispheres (2 from the left and 2 from the right) per region (CMM, NCM, hippocampus) per subject. For each hemisphere of each region, we used the combined number of Zenk-IR cells at 1.00 and 1.06 mm caudal to coordinates zero for further analysis (see Fig. 4b). Image analysis was carried out semiautomatically with a PC-based system equipped with the KS400 version 3.0 software (Carl Zeiss Vision, Oberkochen, Germany), which is described in detail in a previous report^39^.

### Statistical analysis

The data were transformed before statistical analysis to satisfy the assumptions of the parametric tests (see the supplementary information for details on data transformation). In experiment 1, we conducted repeated-measures analyses of variance (ANOVAs) with individual subject as a repeated factor and one-sample *t* tests (two tailed). To test for sex difference in vocal behaviour in experiment 2, we conducted a repeated-measures ANOVA with Sex as independent factor and different months (Time) as repeated factor. The sample size was unbalanced between males and females in behavioural data of experiment 2. This unbalance presents problems for a two-way factorial design^65^. Using the behavioural data used in repeated-measures ANOVAs, we conducted Bayesian analyses with hierarchical regression models specifically designed for unbalanced data^66^. These analyses yielded results qualitatively similar to those reported in the Results section (see the supplementary information for the results of Bayesian analyses). Males and females were analysed separately for each brain region using repeated measures ANOVAs with the Stimulus (that is, silence, mate) as independent factor and Hemisphere (that is, left, right) as repeated factor. For the mate-specific behaviour, we combined data from the two sexes for each pair, and then conducted repeated-measures ANOVAs with individual pair as a repeated factor. Levels of significance were set at P < 0.05. Data were analysed using StatView version 5 (SAS Institute, Inc., Carey, NC, U.S.A.).

### Ethics statement

All experimental procedures were in accordance with Japanese law, and approved by the Animal Experiments Committee of Japan Women’s University (Permit Number: II 07–13).

## Additional Information

**How to cite this article**: Eda-Fujiwara, H. *et al.* Sex differences in behavioural and neural responsiveness to mate calls in a parrot. *Sci. Rep.* **6**, 18481; doi: 10.1038/srep18481 (2016).

## Supplementary Material

Supplementary Information
