# Supplementary material for: Sex differences in behavioural and neural responsiveness to mate calls in a parrot
Source: Sci Rep. 2016 Jan 4;6:18481. doi: 10.1038/srep18481 (PMC4698741; doi:10.1038/srep18481)
Supplement: Supplementary Information [file srep18481-s1.pdf]

# **Sex differences in behavioural and neural responsiveness to mate calls in a parrot**

Hiroko Eda-Fujiwara, Ryohei Satoh, Yuka Hata, Marika Yamasaki, Aiko Watanabe, Matthijs A. Zandbergen, Yasuharu Okamoto, Takenori Miyamoto & Johan J. Bolhuis

## **SUPPLEMENTARY INFORMATION**

## **Methods**

**Housing.** Males and females were acquired from different breeders and were housed in separate rooms, with each bird in an individual wire-mesh cage (22 x 17 cm and 33 cm high), so that birds were unfamiliar with the birds of the opposite sex prior to the study. The individual cages were fitted with wooden perches, but not with nestboxes. Each bird could hear and see other birds of the same sex in the room. Each pair was housed in a wire-mesh cage (45 x 45 cm and 57 cm high) with a wooden nestbox (18.5 x 13.5 cm and 13 cm high) and a wooden perch for 35 days. Pairs were prevented from seeing other pairs in the same room by means of wooden barriers; however, they were in vocal contact with other pairs.

**Behavioural observations.** To assess the development of pair bonds, we performed continuous 120 min observations between 0900 and 1100 hours on days 2, 15, and 35 after the pairs were placed into breeding cages, as described preveiously (Eda-Fujiwara *et al.* 2011). Allopreening, in which one individual cleans the other’s feathers with its beak, is one of the behaviours associated with pair bonding in the budgerigar; it occurs more frequently with mates than with nonmates (Trillmich

1976; Zocchi & Brauth 1990). Courtship feeding, in which a male feeds a female in beak-to-beak contact, is also shown more often to mates than to nonmates (Trillmich 1976). We, following Trillmich (1976), defined allopreening and courtship feeding as the mate-specific (or partner-specific) behaviour and used this behaviour to assess the development of pair bonds. Individual one-zero records of allopreening and courtship feeding were taken every 3 min (i.e. 40 records per day per subject).

**Recording of contact calls.** During recording sessions prior to pairing, two birds at a time were placed in a soundproof chamber to encourage the birds to vocalise. In experiment 1, each male was placed with one of three males, which were only used for the recording sessions and were socially unfamiliar to all of the subjects used for pairing. In experiment 2, each subject (male or female) was placed with one of the birds of the same sex used in experiment 2. The soundproof chamber contained two cages separated by a transparent, 10 mm thick Plexiglas divider. One microphone (ECM-T150; Sony Corp., Tokyo, Japan) was placed on each side of the divider and connected to a digital audio recorder (PCM-D50; Sony Corp., Tokyo, Japan). Sounds from both microphones were stereo-recorded so that sound intensities could be compared between the two channels and thereby caller identity could be determined.

The vocalisations recorded during the present study were filtered (band-pass filter: 1.5-5.5 kHz) and displayed as spectrograms (fast Fourier transformation, FFT, size: 256, temporal resolution 3 ms) using the sound analysis software Avisoft SASLab Pro (Avisoft Bioacoustics, Berlin, Germany). The methods used to classify contact calls were similar to those described in previous studies (Farabaugh *et al*. 1994; Eda-Fujiwara *et al*. 2011). Contact calls of each bird before

pairing were classified into call types by an observer naïve to the identity of the birds. Of all call types for each bird, one call type occurs most frequently and is therefore termed 'the dominant contact call'. We determined this call for each bird. In our previous study (Eda-Fujiwara *et al.*, 2011), we conducted both of the two analyses (i.e., visual inspection and cross-correlation) for the same data set to assess similarity/dissimilarity between contact calls. In comparisons rated as 1 on our ordinal scale of similarity, cross-correlation values were less than 0.75, the threshold, which has been used to decide whether two calls are similar in the budgerigar. Then, we can use visual inspection instead of cross-correlation.

**Construction of stimuli.** Each recorded mate's call used in the preference tests was randomly chosen from recordings of the bird's dominant call type made before pairing. To avoid pseudoreplication (Kroodsma 1989), for experiment 1, we prepared calls from 10 females that were unfamiliar to the males; these recordings had been made before pairing in previous pairing experiments using different birds from those used in the present study. Each of the 10 unfamiliar calls used in experiment 1 was chosen from the dominant call type of each unfamiliar female. Similarly, for experiment 2, we prepared 24 unfamiliar calls (12 male calls and 12 female calls).

**Behavioural analyses.** In experiment 1, each male was separated from his mate and placed in an individual cage in a sound-attenuating chamber for at least 1 h before the start of stimulus presentation at m0. The sound-attenuating chamber was equipped with two speakers (AS-5; Kenwood Corp., Tokyo, Japan) placed at either end of the cage and connected to a digital audio recorder (PCM-D50; Sony Corp., Tokyo, Japan). Each speaker was assigned one of the two

stimulus calls. Calls were broadcast at a peak value of 80-85 dB SPL (NA-14 Sound Level Meter; Rion Corp., Tokyo, Japan; A-weighting, slow response), which was measured at the end of the cage. The two calls (a mate's and an unfamiliar call) in a stimulus pair were adjusted to have the same peak amplitude. The vocal activity of the male was recorded with a digital audio recorder (PCM-D50; Sony Corp., Tokyo, Japan), and we counted the calls produced during the broadcast of the stimuli. After the 2 h trial, the bird was kept in the sound-attenuating chamber and presented with the same stimuli in a 2 h trial on the next day. In the m0 test, individual males were subjected to two 2 h trials, with each call being broadcast from the alternate speaker in the two trials to control for possible side preferences. Data from the two trials for each bird were pooled for the test at m0. We used the same procedure for the other two tests (that is, at m1 and m5). The procedure in experiment 2 was identical to that in experiment 1.

**Data transformation**

The behavioural data were arcsine transformed for the preference ratio and log transformed for the total number of calls before statistical analysis to satisfy the assumptions of the parametric tests. All Zenk data were log transformed before statistical analysis. For the mate-specific behaviour associated with pair bonding, we log transformed before statistical analysis.

**Bayesian analyses**

We conducted three behavioural tests at days 38.5 $\pm$ 3.5 before separation and at days 3.5 $\pm$ 3.5 and 35 $\pm$ 5 after separation, which are designated 'pre', 'post(m0)' and 'post(m1)', respectively in experiment 2(see Fig. 3a). For these behavioural data we performed Bayesian analyses with

hierarchical regression models, to examine whether the call-response ratio was determined by each of two variables: (1) sex; (2) separation time. We introduced three dummy variables $X_1$, $X_2$, and $X_3$.

$X_1$ = 0 (female), 1 (male)

$X_2$ = 0 (pre), 1 [post(m0)]  (or 0 [post(m0)], 1 [post(m1)])

$X_3 = X_1 X_2$

We fitted the model

$$Y_{ij} = \mu_i + \alpha X_1 + \beta X_2 + \gamma X_3 + \varepsilon$$

where $Y_{ij}$ is arc-sin transformed value of the call-response ratio that bird *i* shows at time *j*.

*j* = pre, [post(m0)]  (or [post(m0)], [post(m1)])

$$\mu_i \sim N(\mu, \alpha^2{}_\mu), \quad \mu \sim U(-A, B), \quad \sigma_\mu \sim U(0, B)$$

$$\alpha \sim U(-A, B), \quad \beta \sim U(-A, B), \quad \gamma \sim U(-A, B), \quad \sigma_\varepsilon \sim U(0, B)$$

For each regression coefficient, we calculated the 95% Credible Interval, corresponding to the confidential interval in the standard statistical method. If the Credible Interval of a regression coefficient (e.g. the coefficient $\alpha$ showing the effect of Sex) does not include zero (the value in our null hypothesis), it indicates that the variable (Sex in this example) has a significant effect on the call-response ratio. The coefficient $\beta$ and $\gamma$ shows the effect of Time, and the interaction between

Sex and Time, respectively.

## Results

### Sex differences in behavioural responsiveness to mate calls.

*Experiment 2.* We performed Bayesian analyses with hierarchical regression models to investigate possible sex differences in the call-response ratio for mate calls and to address the problem which unbalanced data presents for the two-way factorial ANOVA (Table S1). In the analysis to test for the effect of factors of Sex and Time (i.e. before the pairing period and 0 month after pair separation), we found a significant positive effect of Time on the call-response ratio for mate calls in females, as the 95% Credible Interval for the regression coefficient for Time did not include zero. This indicates the call-response ratio for mate calls was higher at 0 month after separation than before the pairing period in female budgerigars. In males, the call-response ratio for mate calls did not change between these two time points, as the 95% Credible Interval for $\beta+\gamma$, which represents increase of the call-response ratio at 0 month in males, included 0.

In the analysis to test for the effect of factors of Sex and Time (i.e. 0 and 1 months after pair separation), we found a significant negative effect of Sex and a significant negative effect of Time on the call-response ratio for mate calls, as the 95% Credible Intervals for the regression coefficients did not include zero. There was not a significant interaction between Sex and Time, as the Credible Interval contained zero. This indicates stronger behavioural responsiveness for mate calls in females than in males.

## References

Eda-Fujiwara, H., Kanesada, A., Okamoto, Y., Satoh, R., Watanabe, A. & Miyamoto, T. Long-term maintenance and eventual extinction of preference for a mate's call in the female budgerigar. *Anim. Behav*. **82**, 971-979 (2011).

Trillmich, F. Spatial proximity and mate-specific behaviour in a flock of budgerigars (*Melopsittacus undulatus*; Aves, Psittacidae). *Z. Tierpsychol.* **41**, 307-331 (1976).

Zocchi, D.C. & Brauth, S.E. An experimental study of mate directed behaviour in the budgerigar *Melopsittacus undulatus*. *Bird Behaviour* **9**, 49-57 (1990).

Farabaugh, S.M., Linzenbold, A. & Dooling, R.J. Vocal plasticity in budgerigars (*Melopsittacus undulatus*): Evidence for social factors in the learning of contact calls. *J. Comp. Psychol*. **108**, 81-92 (1994).

Kroodsma, D.E. Suggested experimental design for song playbacks.*Anim. Behav*. **37**, 600-609 (1989).

Table S1. Results of Bayesian analyses with hierarchical regression models.

| | 95% credible interval of the regression coefficient |
|---|---|
| Effect of factors on the call-response ratio for mate calls at pre and post(m0) | |
| Sex ($\alpha$) | [-7.28, 7.83] |
| Time ($\beta$) | [4.77, 16.78] |
| Sex*Time ($\gamma$) | [-15.79, -0.61] |
| Effect of factors on the call-response ratio for mate calls at post(m0) and post(m1) | |
| Sex ($\alpha$) | [-18.33, -4.30] |
| Time ($\beta$) | [-19.36, -4.05] |
| Sex*Time ($\gamma$) | [-3.83, 14.20] |
